# Mapping of the methylation pattern of the hMSH2 promoter in colon cancer, using bisulfite genomic sequencing

**DOI:** 10.1186/1477-3163-5-22

**Published:** 2006-08-15

**Authors:** Hua Zhang, Wei-ling Fu, Qing Huang

**Affiliations:** 1 Department of Clinical laboratory, Southwest Hospital, Third Military Medical University, Chongqing, 400038, China

## Abstract

The detailed methylation status of CpG sites in the promoter region of hMSH2 gene has yet not to be reported. We have mapped the complete methylation status of the hMSH2 promoter, a region that contains 75 CpG sites, using bisulfite genomic sequencing in 60 primary colorectal cancers. And the expression of hMSH2 was detected by immunohistochemistry. The hypermethylation of hMSH2 was detected in 18.33% (11/60) of tumor tissues. The protein of hMSH2 was detected in 41.67% (25/60) of tumor tissues. No hypermethylation of hMSH2 was detected in normal tissues. The protein of hMSH2 was detected in all normal tissues. Our study demonstrated that hMSH2 hypermethylation and protein expression were associated with the development of colorectal cancer.

## Background

Methylation mapping is a particularly useful method for determining the methylation profile of a gene[1], and for some genes, including RASSF1A [2], E-cadherin [3], p16[4], cyclooxygenase-2 [5] and the estrogen receptor [6], the methylation pattern of the promoter region has been thoroughly examined. However, despite the importance of methylation mapping in investigating profiles of the promoter region of hMSH2 have yet to be examined. Hence, to examine the methylation profiles of 75 CpG sites in the 979-bp promoter region of the human hMSH2 gene, we performed conversion-specific bisulfite genomic sequencing in 60 primary colorectal cancers. Furthermore, the expression of hMSH2 gene was examined using immunohistochemistry.

## Materials and methods

### Subjects

Primary colon cancer tissue was obtained by surgical resection from 60 patients (37 male, 23 female; median age of 56 years, range 24–83 years) with sporadic colorectal cancer. All of these patients corresponding non cancer colon tissue was also obtained from a tumour-free location, which was at least 5 cm distant from the tumour and which was confirmed to be without any tumour cell infiltration by histology. Immediately after surgery, tissue samples were put in liquid nitrogen and stored at -80°C until use.

### DNA extraction and bisulfite genomic sequencing

Genomic DNA of all samples was extracted from the tissues using QIAamp^® ^DNA Mini Kit (Qiagen, Inc., Germany), according to the manufacturer's instructions. Bisulfite modification of the genomic DNA was carried out using DNA Modification Kit (ZYMO Research, USA). The modified DNA was amplified by PCR, using the two sets of primers shown in Table 1. The primers were designed for the hMSH2 promoter and exon1 (GenBank accession number U23824, U41206) [25] using MethPrimer, a program for designing bisulfite-conversion-based methylation PCR primers [26]. These primers were specific for the modified DNA, but did not contain any CpG sites in their sequence, and therefore both methylated and unmethylated DNA can be amplified by the same primer sets. Each PCR mixture contained genomic DNA, 1.25 units TaKaRa Ex Taq HS (Takara, Tokyo, Japan), 1X Ex Taq buffer, 2 mM deoxynucleotide triphosphate mixture, and 50 pmol sense and antisense primers in a volume of 25 μl. The PCR conditions were 94°C for 5 min, 35 cycles of 94°C for 30 s, 55°C for 30 s, and 72°C for 60 s, With a final extension reaction at 72°C for 5 min. The PCR products were sequenced on an ABI automated sequencer 3100 with Dye terminators (ABI, USA).

**Table 1:** Primers used for bisulfite genomic sequencing

Name	Sequence	Number of CpG sites in PCR product	Size (bp)
MSH2-1F	AGGGGTTTTAAGTTTTGTAGTTGAG	46	520
MSH2-1R	CCATATACTTAATCACCCCCTAAAT		
MSH2-2F	TTAAGATTTAGGGGGTGATTAAGTA	29	459
*MSH2-2R*	TATCATAAAAAAATCTCCTAAACCC		

### Immunohistochemistry

Deparaffinized serial sections were cut at 4 μm for immunohistochemistry and placed on Superfrost Plus glass slides. Prior to immunostaining, sections were deparaffinized in xylene and rehydrated in an alcohol series. Immunostaining was performed with hMSH2 antibody (CALBIOCHEM, USA).

Incubation with the primary antibodies was performed in a moist chamber at 37°C for 1 h. After incubation with the secondary antibody (30 min, room temperature; Dako, Denmark). Immunohistochemical staining was performed using EnVision™ Detection Kit(Dako, Denmark). Evaluation of the slides was performed by an experienced pathologist who was blinded to the results of the methylation analysis.

### Statistical analysis

Statistical analysis was performed using a X^2 ^test for comparison of the overall methylation level. A probability of P < 0.05 was considered to be statistically significant.

## Results

### Analysis of hMSH2 methylation

**Figure 1:**
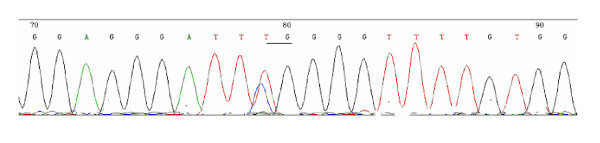
**Examples of bisulfite genomic sequencing chromatography**. DNA was amplified and sequenced using each primer set on an ABI automated sequencer with dye terminators. After bisulfite treatment, unmethylated cytosine was converted to thymine. TG is a partial methylation point.

Bisulfite DNA sequencing was carried out to assess the extent of CpG island methylation in the hMSH2 gene promoter in carcinoma cells and normal colonic cells. Results was shown in Table 2. Examples of such sequencing are presented in Fig 1: note that after bisulfite treatment, unmethylated cytosine was converted to thymine. The hypermethylation of hMSH2 was detected in 18.33% (11/60) of tumor tissues. No hypermethylation of hMSH2 was detected in normal tissues.

**Table 2:** methylation of hMSH2

**Group**	**Cases**	**Methylation**	**Unmethylation**	***P *Value**
Age				
< 50	17	3	14	
≥50	43	8	35	P > 0.05
Sex				
Male	37	7	30	
Female	23	4	19	P > 0.05
Position				
Colon cancer	28	5	23	
Rectal cancer	32	6	26	P > 0.05
Ducks stage				
A,B	42	2	40	
C,D	18	9	9	P < 0.01
Histodifferentiation degree				
Well	13	1	12	
Moderate, poor	47	10	37	P < 0.01
Lymph node and distant metastasis				
Yes	18	8	10	
*NO*	42	3	39	P < 0.05

### Immunohistochemistry

**Figure 2:**
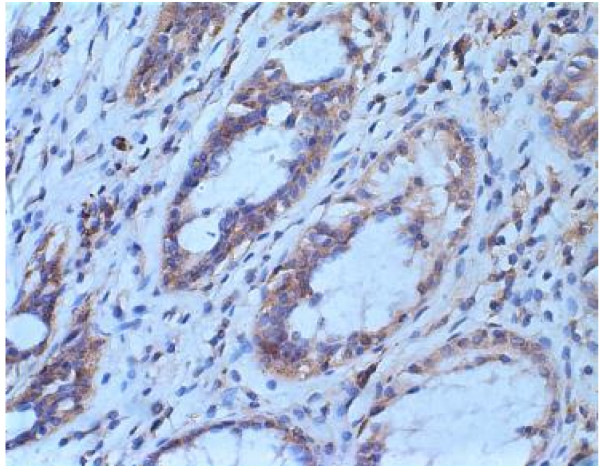
Normal cells showed stable expression.

**Figure 3:**
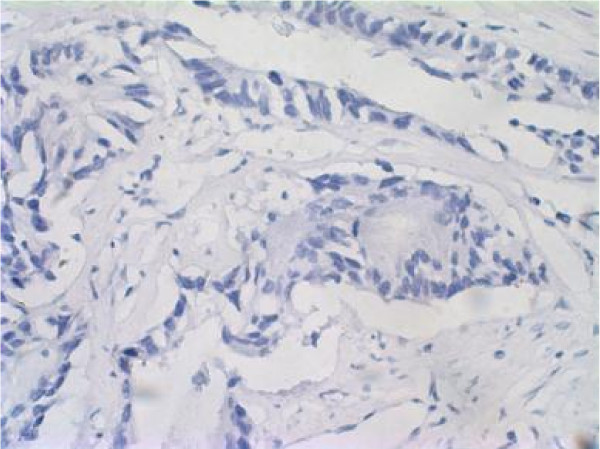
cancer cells did not show immunoreactivity.

The results of immunohistochemistry are shown in Fig. 2 and Fig. 3. The protein of hMSH2 was detected in 41.67% (25/60) of tumor tissues. The protein of hMSH2 was detected in all normal tissues.

## Discussion

Methylation mapping is one of the most useful methods available for determination of the methylation profiles of a promoter region. In the present study, we were able to achieve complete methylation mapping of the hMSH2 promoter region, using primers obtained from Methprimer software. This program is useful for designing specific PCR primers that can perform selective amplification of modified DNA only [7].

We results showed: the hypermethylation of hMSH2 was detected in 18.33% (11/60) of tumor tissues. The protein of hMSH2 was detected in 41.67% (25/60) of tumor tissues. No hypermethylation of hMSH2 was detected in normal tissues. The protein of hMSH2 was detected in all normal tissues. Our study demonstrated that hMSH2 hypermethylation and protein expression were associated with the development of colorectal cancer.

In studies using bisulfite genomic sequencing and methylation specific PCR, contamination with stroma may yield false positive results. Thus, to eliminate stromal cells from the samples, studies using microdissected specimens are recommended for future studies.

In conclusion, this study has clarified the detailed methylation status of wide area of the hMSH2 promoter region in colon cancer. The results suggest that methylation of certain CpG sites may play a particularly important role in the regulation of hMSH2 gene transcription. Primers used for future MSP analysis can be designed on the basis of these detailed sequencing data, and hence our mapping data provide an important basis for further studies of methylation-regulated hMSH2 inactivation.
